# Mesenchymal Stem Cells and Purinergic Signaling in Autism Spectrum Disorder: Bridging the Gap between Cell-Based Strategies and Neuro-Immune Modulation

**DOI:** 10.3390/biomedicines12061310

**Published:** 2024-06-13

**Authors:** Agata Wikarska, Kacper Roszak, Katarzyna Roszek

**Affiliations:** Department of Biochemistry, Faculty of Biological and Veterinary Sciences, Nicolaus Copernicus University in Torun, Lwowska 1, 87-100 Torun, Poland; 302253@stud.umk.pl (A.W.); 302246@stud.umk.pl (K.R.)

**Keywords:** autism spectrum disorder, neuroinflammation, neuro-immune modulation, purinergic signaling, cell-based therapy

## Abstract

The prevalence of autism spectrum disorder (ASD) is still increasing, which means that this neurodevelopmental lifelong pathology requires special scientific attention and efforts focused on developing novel therapeutic approaches. It has become increasingly evident that neuroinflammation and dysregulation of neuro-immune cross-talk are specific hallmarks of ASD, offering the possibility to treat these disorders by factors modulating neuro-immunological interactions. Mesenchymal stem cell-based therapy has already been postulated as one of the therapeutic approaches for ASD; however, less is known about the molecular mechanisms of stem cell influence. One of the possibilities, although still underestimated, is the paracrine purinergic activity of MSCs, by which stem cells ameliorate inflammatory reactions. Modulation of adenosine signaling may help restore neurotransmitter balance, reduce neuroinflammation, and improve overall brain function in individuals with ASD. In our review article, we present a novel insight into purinergic signaling, including but not limited to the adenosinergic pathway and its role in neuroinflammation and neuro-immune cross-talk modulation. We anticipate that by achieving a greater understanding of the purinergic signaling contribution to ASD and related disorders, novel therapeutic strategies may be devised for patients with autism in the near future.

## 1. Introduction

Autism spectrum disorder (ASD) covers several neurodevelopmental disorders that, to varying degrees, affect the patient’s behavior, ability to communicate, and social interaction. ASD manifests itself early in life and often lasts a lifetime, but the severity of symptoms varies from person to person. There has been an upward trend in the prevalence of ASD over the decades, with a rapid increase within the last few years. According to the Centers for Disease Control and Prevention (CDC) report, in the United States, ASD was diagnosed in 1 in 59 eight-year-olds in 2014, 1 in 54 in 2016, 1 in 44 in 2018, and 1 in 36 in 2020 [1,2]. The World Health Organization (WHO) statistics show that within 16% of the global child population, the prevalence of ASD is approximately 0.76% [3]. Accordingly, the increasing occurrence highlights a growing need for resources to provide care for this population of children. It is estimated that by 2025, in the US, medical, non-medical, and productivity costs spent on ASD individuals will reach 461 billion USD [4]. Furthermore, these patients are exposed to high stress due to difficulties in social interactions or transitioning from childhood to adulthood (e.g., problems in education or employment struggle), which can lead to anxiety and depression, and for that reason, require more support and mental health services [5,6].

There are currently no approved therapies that address the core symptoms of autism. Thus, there is a keen interest in exploring novel therapeutic approaches to help patients with ASD and comorbidities. In addition to behavior or nutritional therapies, cell-based therapies are also offered. The most often used cells are mesenchymal stem (stromal) cells (MSCs). The first discovery of MSCs was made in the mid-1960s when Alexander Friedenstein stated that bone marrow is a reservoir of not only hematopoietic stem cells but also stem cells of mesenchymal origin [7,8]. The term “Mesenchymal Stem Cells” was not proposed until 1991 by Caplan, who is currently suggesting renaming these cells “Medicinal Signaling Cells” due to their paracrine activity and immunomodulatory influence [7]. As of 2023, over 1400 clinical studies involving MSCs as a potential treatment have been registered to date, according to ClinicalTrials.gov (records for the term: “Mesenchymal Stem Cells”) [9]. The basis of MSCs’ mechanism of action has already been recognized—they act through cell-to-cell interaction or the release of various factors, e.g., cytokines, nucleotides and nucleosides, and growth factors, secreted into the microenvironment or encapsulated in extracellular vesicles [10]. However, the exact mode of action of MSC-mediated therapy in different diseases is still not fully recognized and requires further research. Cell-based interventions are also tested in clinical trials for many neurological diseases including, but not limited to, cerebral palsy, hypoxic–ischemic encephalopathy, severe traumatic brain injury, spinal cord injury, stroke, and ASD [11]. In most studies, cells are delivered intravenously and claimed to modulate neuroinflammation as one of their mechanisms of action, but none of these are currently fully substantiated.

The purinergic signaling system is the oldest evolutionary transmitter system that utilizes extracellular purines and pyrimidines, ATP and adenosine (Ado), in particular, as chemical messengers [12]. The binding of extracellular nucleotides or nucleosides to their specific receptors triggers numerous signaling pathways and regulates a wide array of physiological and pathophysiological processes [13]. Purinergic receptors, first discovered in 1976, have been divided into two families—P1 and P2 receptors [12,14,15]. The family of P1 receptors is classified into four subtypes, A1, A2A, A2B, and A3, each encoded by a different gene [12,15]. All subtypes are G-protein-coupled receptors that bind adenosine and couple to adenylate cyclase: A1 and A3 receptors negatively and A2A and A2B receptors positively [12,13,14]. Numerous research studies have proved the role of P1 receptors in key processes, such as the modulation of immune and central nervous system functions [12,14]. Furthermore, studies point out the importance of A1 and A2A receptors in the development of psychiatric disorders such as depression and bipolar disorder [14]. A1R (A1 receptor) is associated with neuroprotection, while A2AR takes part in neuroinflammatory processes [14]. P2 is a larger family of receptors classified into two subfamilies, P2X and P2Y [12,13,14,15,16]. In mammals, seven P2X receptors and eight P2Y receptors have been currently described [12]. P2Y are a subfamily of G-protein-coupled receptors, while P2X are trimeric ligand-gated ion channel receptors [12,13,14,16]. They are permeable to sodium, potassium, and calcium in response to extracellular ATP [13,16]. P2X receptors are widely expressed in the mammalian brain in neuronal and glial cells [15,16]. The most well-known receptor is P2X7, expressed, among others, in monocytes, macrophages, neutrophils, lymphocytes, and mast cells [17]. Its activation by ecto-ATP is largely associated with key inflammatory responses and is implicated in various neurological disorders [14,15,16].

Apart from the receptors, another key component of purinergic signaling is the set of membrane-bound and soluble enzymes breaking down nucleotides and nucleosides in the extracellular environment [14,18]. The main role of those enzymes includes regulating the duration of action of the agonist on the receptor, as well as providing the products of hydrolysis as ligands for the purinoreceptors [18]. The main purine-hydrolyzing enzymes are ectonucleoside triphosphate diphosphohydrolases (E-NTPDases), ectonucleotide pyrophosphatase/phosphodiesterases (E-NPPs), alkaline phosphatases (APs), ecto-5′-nucleotidase (E-5′-NT), and adenosine deaminase (ADA) [14]. Some of them, including NTPDase1 (otherwise known as CD39 antigen) and E-5′-NT (known as CD73), are described to play a crucial role in inflammatory processes [13].

We anticipate that by achieving a greater understanding of the pathophysiology and pathogenic mechanisms involved in ASD and related disorders, novel therapeutic strategies may be devised for patients with autism in the near future. Some of them can be targeted toward the elements of the purinergic signaling pathway.

## 2. What Is Autism Spectrum Disorder?

The first written records of autism date back to 1943, when psychiatrist Leo Kanner described it as a disorder associated with high sensitivity of children to environmental changes affecting young patients’ ability to relate to others [19]. Autism spectrum disorder (ASD) is a highly heritable neurodevelopmental ailment that is characterized by abnormalities in social interactions, which can be expressed by verbal and nonverbal communication pathology (e.g., mismatched voice intonation, lack or reduction of sharing interest and emotions, avoiding eye contact, incomprehensible gestures), inability or difficulties in developing and maintaining relationships, and the problem with understanding personal space. In addition to social impairment, ASD patients represent restricted, repetitive patterns of behavior, activities, or interests that manifest themselves through specific, repetitive movements (e.g., motor stereotypes, echolalia), restricted interests (e.g., strong interest in a particular topic, attachment to unusual objects), abnormal sensitivity to environmental stimuli (e.g., light fascination, inappropriate reaction to specific sounds), and problem with interrupting routine activities (e.g., presence of greeting patterns, stress during minimal changes) [19,20,21]. The above-mentioned manifestation of ASD was published in the *Diagnostic and Statistical Manual of Mental Disorders* (DSM), *Fifth Edition*, currently the main textbook used by psychotherapists for the diagnosis and classification of ASD [20]. Those two main criteria are also used to determine the severity of the disease and to classify the patient into one of three groups of requirement assistance in everyday life [20].

Aside from those symptoms, many ASD individuals also experience language and intellectual impairment, which, combined with a medical diagnosis of other genetic, neurodevelopmental, and mental conditions, as well as environmental factors that increase the risk of disease, are used for better interpretation of diagnostic features [20]. The diagnosis of ASD should be evaluated by an interdisciplinary team of experts or, when the situation does not allow for such a committee, a specialist with experience in determining psychiatric disorders. The process of establishing diagnosis should be based on the patient’s history and determination of behavioral impairments leading to rejection of diseases with similar symptoms to ASD, establishing of possible comorbid conditions, and classification of the level of functioning [19]. According to the DSM-V document, diseases such as Kanner’s autism, Asperger’s disorder, early infantile autism, childhood autism, atypical autism, high-functioning autism, childhood disintegrative disorder, and pervasive developmental disorder not otherwise specified are now included in the definition of spectrum and are not treated as separated subtypes [20]. 

Scientific articles constantly mention newer and newer risk factors of ASD. Among them, it is possible to distinguish those pre- and postnatal factors, including genetic factors, maternal body influence during pregnancy, environmental toxic pollutants, and drug supplementation. Due to this broad spectrum of factors associated with ASD pathogenesis, the establishment of a direct cause of this disorder seems to be barely possible [22].

## 3. Etiology of Autism Spectrum Disorder

### 3.1. Environmental Factors

During prenatal and early postnatal life, children are exposed to a wide range of environmental risk factors. In the period of the most intense human development, such exposition can lead to drastic developmental abnormalities, causing ASD [23]. Therefore, many compounds to which we are exposed in everyday life, such as air pollutants, can lead to neurodevelopmental disorders when they affect pregnant women or infants [24,25].

Maternal health conditions during pregnancy influence the proper neurodevelopment of the fetus. Research shows that obesity, which occurs in 20% of the adult worldwide population, during pregnancy can increase the risk of ASD by up to 36%. In addition, increased body weight is also associated with the presence of diabetes [26]. According to one of the cohort studies that included 419,425 children, the unadjusted average annual ASD incidence rates per 1000 children were as follows: for type I diabetes, 4.4; for type II diabetes, 3.6; for gestational diabetes by 26 weeks, 2.9; for gestational diabetes after 26 weeks, 2.1; and for no diabetes, 1.8, respectively [27].

Among medications, antidepressants and antiepileptic drugs are considered as possibly harmful for fetuses during maternal administration. Mostly because of the ability to cross the placenta and blood–brain barrier and their presence in breast milk [28,29]. There is some evidence that medications used by mothers in the treatment of psychiatric disorders during pregnancy increase the risk of ASD by 68% [28]. Animal model studies have shown that selective serotonin reuptake inhibitor (SSRI) antidepressants may affect brain development [30]; however, studies on the correlation between taking SSRI antidepressants during pregnancy and possible prenatal ASD induction are inconsistent and still require more research [29,30]. Several lines of evidence indicate the association of valproate (medicine for epilepsy) use during pregnancy with an increased risk of ASD [31,32]. 

In public opinion, there is still a conviction that vaccines can cause neurodevelopmental disorders. Everything started when Wakefield et al. published an article about MMR vaccines as a risk factor for ASD. It raises the speculation that one of the components of the vaccines—thiomersal, which contains 50% ethylmercury—could affect the functioning and development of the brain. However, Wakefield’s study has been retracted due to data falsification, and later studies rejected the harmfulness of thiomersal [23,33].

### 3.2. Genetic Factors

ASD is characterized by high heredity, which brings the conclusion that genetic factors play a crucial role in the pathophysiology of this disease. The studies comparing the phenotypic profile of 100% similar monozygotic twins and dizygotic twins, which represent 50% of genetic similarity, show that heredity of ASD in monozygotic twins estimates between 60 and 90% and between 5 and 40% in dizygotic twins, which makes ASD one of the most heritable neurodevelopmental disorders [34]. Unfortunately, only approximately 5–10% of all ASD cases co-occur with monogenic disorders [35]. Most of the disorders are associated with a mutation in the sequence of regulators that control the expression of groups of genes, taking part in processes such as chromatin packing, development of an embryo, and synaptic transmission [35,36]. Within them, Rett syndrome and Fragile X syndrome represent the most frequent ones, in which 40% and 25% of patients are co-diagnosed with ASD, respectively [37].

Two groups of genetic aberrations can cause ASD: copy number variants (CNVs) and single-nucleotide variants (SNVs), all of which could be inherited from one or both of the parents or appear de novo [38]. CNVs, compared to large chromosomal aberrations, refer to relatively small DNA regions that undergo duplications or deletions [36]. They are detected in the genome by using microarray techniques that offer high resolution and are able to distinguish abnormalities between ASD and healthy patients [36,37]. All CNVs that have been correlated with ASD are described as rare, highly penetrant genetic variants [39]. There are two groups of CNVs according to the ability to recur: those which are recurrent and those which are not [35]. The type that appears de novo is more frequent (4–10% of ASD patients) than the recurrent one (3%) [40]. De novo CNVs are more often observed in idiopathic ASD amidst simplex families than in multiplex families and those without affected members [40].

About 0.1% of the whole human genome are single nucleotide variations (SNVs) that take part in creating the unique genetic profile of every individual [41]. SNVs can be divided into rare mutations and single nucleotide polymorphisms (SNPs). SNPs refer to variants that occur in at least 1% of the population [35,40]. SNPs usually represent little effects in terms of causing ASD but can group together to form polygenic complexes with additive effects, which highlight the heterogeneous genetic composition of ASD [39]. For analyzing SNPs, scientists have been using Genome-Wide Association Studies (GWAS), which calculate the prevalence of SNPs between large groups of patients with and without ASD [36].

### 3.3. Immune Dysregulation

#### 3.3.1. Prenatal Inflammation

The first mentions of connections between ASD and immune system activation were in the 1960s when the rubella epidemic spread across the U.S. [42,43]. Back then, it was noted that mothers during pregnancy who had undergone rubella infection and gave birth to a child with congenital rubella syndrome had an 8–13% probability of ASD occurrence among their offspring [43]. This and many other studies about viral infections contributed to general speculation that activation of the immune system can affect fetal neurodevelopment [42].

Today, it is well known that maternal immune activation (MIA) caused by infections (viral or bacterial), inflammation, coexisting autoimmune diseases, and stress during the gestation period can induce neuroinflammation and lead to altered fetus neurodevelopment, causing ASD or other neurodevelopmental diseases [detailed information can be found in some recent extensive reviews, e.g., [43,44,45]. During MIA, there is an increased production of proinflammatory cytokines and chemokines due to the activation of immune cells, including T lymphocytes and macrophages [44]. Many of such molecules can cross the immature blood–brain barrier (BBB) of the fetus along with increasing its permeability, eventually causing detrimental effects on the still-developing brain [46]. Among all cytokines participating in MIA, IL-6 and IL-17A are the most often mentioned and widely described ones [43,47].

Another key factor in MIA is maternal antibodies, which normally supplement the immature fetal immune system [48]. These antibodies, in pathological conditions, can bind to epitopes of fetal proteins that are important in proper neurodevelopment, eventually leading to ASD [46,48,49]. It is estimated that one in ten mothers with ASD children produces anti-fetal antibodies [49]. Amidst the identified target fetal brain proteins of immunoglobulins are Y-box binding protein 1 (YBX1), collapsin response mediator proteins 1 and 2 (CRMP1/2), neuron-specific enolase (NSE), guanine deaminase or cypin (GDA), stress-induced phosphoprotein-1 (STIP1), and lactate dehydrogenase A and B (LDHA/B) [46,48,49].

#### 3.3.2. Postnatal Inflammation

Besides the maternal immune influence on the fetus during gestation, after-birth immune dysregulations have also been reported. There is still a growing list of evidence that these abnormalities are mostly directed toward proinflammatory responses rather than anti-inflammatory ones [50]. Interestingly, it correlates with findings that within ASD patients’ families, autoimmune diseases, like diabetes mellitus type 1 or rheumatoid arthritis, are more frequently noted [51]. In the course of ASD, neuroinflammation is mainly observed in such brain areas as the cerebral cortex, white matter, and cerebellum [22]. Based on clinical studies, neuroinflammatory processes observed in patients with ASD are often chronic. Various signs of inflammation in the brain have been described in all age groups in the systematic screening of postmortem samples [22].

Cytokine profiles reflect the differences between ASD individuals and those from control groups. The abnormalities were described in the brain, cerebrospinal fluid, and peripheral blood in mouse models but also in ASD-diagnosed patients [52]. Proinflammatory cytokines being upregulated during ASD include IL-1β, IL-6, IL-17, IL-18, IL-33, TNF-α, IFN-γ, and many other [50,52,53,54]. Moreover, ASD patients have reduced expression of TNF and hnRNPL-related immunoregulatory lincRNA (THRIL) gene, the negative regulator of TNF-α [50]. Cytokine dysregulation during ASD is also strongly associated with abnormalities at the cellular level, e.g., the structure of neurons, synapse formation, the ability to migrate, and adhesion are often impaired [50,53].

A considerable imbalance in the number and properties of immune cells in ASD patients is also a hallmark of postnatal inflammatory processes. Dysfunctions concern monocytes [55,56], T lymphocytes [57,58,59,60,61], microglia [62,63], and astrocytes [64,65]. 

Microglia, as the key cells of the brain’s immune system, can be involved in neuroprotection or neuroinflammation, depending on local signals [66]. Similarly, two phenotypes of astrocytes have been identified—A1, which is neurotoxic, and A2, which shows neuroprotective functions [63,64]. In ASD, communication between microglia and astrocytes is a key process regulating neuroinflammation [63]. LPS-activated microglia switch to an A1 neurotoxic phenotype in astrocytes. Astrocytes further activate microglia and regulate their function. Regulation is mediated through the release of ATP as well as through ORM2 (orosomucoid-2), an acute-phase protein expressed by astrocytes and blocking the CCR5 (C-C chemokine receptor 5) present in microglia [63,67]. All these processes require fine-tuned regulation. 

## 4. How Does Purinergic Signaling Affect Neuroinflammation?

The most well-known mediators of inflammation among purines are ATP and Ado [14]. In physiological conditions, both are present in the extracellular environment in low nanomolar concentrations, but in the situation of distress, hypoxia, or inflammation, their levels rise to micromolar concentrations [17,68,69,70,71]. Both molecules are recognized to act oppositely—ATP displays pro-inflammatory effects, while Ado functions as an anti-inflammatory molecule [14].

ATP is known as an excitatory neurotransmitter, co-transmitter, and neuromodulator [14,69]. It affects several kinds of immune cells, making them alert to stressful situations, e.g., cell damage, and inducing inflammatory processes [14,69]. Accumulation of ATP triggers the recruitment of antigen-presenting cells at the site of inflammation [14]. It also influences immune cells’ differentiation and migration [69,71]. Increased ATP level promotes the formation of oxygen and nitrogen free radicals [71]. The effects of ATP are mediated by P2 receptors; therefore, cells expressing the mentioned receptors at a high level are more sensitive to ATP’s effects [16]. Previous studies have described P2Y1R, P2Y2R, P2Y6R, P2X4R, and P2X7R as the most implicated in inflammation processes in the CNS, although the exact signal transduction mechanisms are still in need of further research [71]. From the whole family of P2X receptors, which are activated by extracellular ATP, the P2X7 receptors require the most attention due to their strong contribution to inflammatory processes. Because of their double activity, as a cationic channel in normal conditions and as a large pore activated by increased concentration of ATP, they are sometimes called “the gatekeepers of inflammation” [72,73]. P2X7R expression has been confirmed in immune cells, including monocytes, macrophages, dendritic cells, NK cells, and T and B lymphocytes, as well as in glial cells such as microglia, astroglia, and oligodendrocytes [72,73]—the important cells in neuroinflammatory processes occurring in neurodegenerative diseases and brain injuries [74,75]. The activation of P2X7 receptors triggers the activation of NLRP3 inflammasome and releases pro-inflammatory cytokines, mostly IL-1β, IL-18, IL-6, and TNF-α, as well as chemokines and growth factors [14,17,71,76]. Signaling via P2X7R also influences the T cells’ activation and modulates the balance between Th17 and Treg lymphocytes [17]. Interestingly, P2X7R itself can trigger the release of intracellular ATP to the extracellular environment, increasing the pool of ecto-ATP [17,76].

Ado, on the other hand, is a well-known immunomodulator that acts through P1 receptors expressed on numerous immune cells [14,70]. Signaling via adenosine initiates mostly immunoprotective processes against cell damage and stressful conditions [70]. The receptors mainly involved in anti-inflammatory processes are A2AR, A2BR, and A3R [71].

Adenosine suppresses neuroinflammation by restraining the proliferation and activation of T cells [14]. Through the activation of adenosine receptors, Ado suppresses the release of pro-inflammatory cytokines such as TNF-α, IL-6, and IL-12, as well as other inflammatory mediators like nitric oxide and MIP-1α, while enhancing the release of anti-inflammatory cytokine—IL-10 [70]. Previous studies have reported that Ado inhibits the production of pro-inflammatory mediators in naive CD4+ T cells and Th1, Th2, and Treg cells [70,77]. Furthermore, adenosine also regulates the activity of dendritic cells and neutrophils, in both of which ARs are expressed [70].

Switching between the pro-inflammatory and anti-inflammatory environment can be achieved via catalyzing the cleavage of ATP to AMP and AMP to Ado through the concerted action of ecto-enzymes, CD39 and CD73, respectively [70,78]. Consequently, these enzymes, as well as other elements of the adenosinergic signaling pathway, are promising targets of anti-inflammatory therapies.

## 5. Functional Attributes of Mesenchymal Stem Cells Revisited

Mesenchymal stem/stromal cells must meet three conditions proposed in 2006 by the International Society of Cellular Therapy (ISCT) to categorize them as MSCs: (1) adherence to plastic surfaces under standard in vitro culture conditions; (2) ability to differentiate into osteoblasts, adipocytes, and chondroblasts; and (3) expression of CD73, CD90, and CD105 molecules but lack of expression of CD45, CD34, CD14, CD11b, CD79a, CD19, and HLA-DR surface molecules [79,80,81,82]. MSCs are present in various adult tissues, which makes them easily accessible and easy to isolate [83]. Bone marrow is the most popular source of MSCs and is used widely in already existing therapies; other sources include adipose tissue, umbilical cord blood and tissue, amniotic fluid, placenta, dental pulp, menstrual blood, synovial membrane, and perivascular cells [22,81,82]. Beyond the MSCs’ role as cells supporting regeneration in various tissues, they also show a unique ability to modulate inflammation, meaning they can activate or suppress the immune system.

### 5.1. Immunomodulatory Properties of MSCs

MSCs affect the mechanisms of innate and adaptive immunity (including both cell-mediated and humoral responses) [80,81]. They modulate the functioning of immune cells through cell-to-cell contact and paracrine actions [79,81]. MSCs present two main phenotypes that change under the influence of the microenvironmental cues activating the Toll-like receptors present in the MSCs’ membrane [81]. The MSC1 phenotype, which is pro-inflammatory, is activated when factors like LPS are present and act on the TLR4 receptors [81]. On the contrary, MSCs can take up anti-inflammatory functions through the MSC2 phenotype, which is influenced by factors like IFN-γ, TNF-α, or poly-I:C acting through TLR3 receptors [81]. Mesenchymal stem cells have already shown successful outcomes in treating various diseases like diabetes, cardiovascular diseases, and graft-versus-host diseases [79]. Because of their attributes, MSCs are also thought to be a therapeutic option in diseases manifested by chronic inflammation, e.g., ASD, Crohn’s disease, multiple sclerosis, systemic lupus erythematosus, rheumatoid arthritis, autoimmune diseases, and inflammatory bowel disease [79,84].

MSC-mediated immunoregulation is realized through many different molecular pathways. This molecular machinery modulates most of the cells of the immune system toward immunosuppression, including those of innate immunity, such as monocytes, macrophages, NK cells, dendritic cells, and neutrophils, and adaptive immunity, such as T cells and B cells. MSC anti-inflammatory activity downregulates the whole range of cytokines essential for proper inflammatory response, including IL-1β, IL-3, IL-6, IL-7, IL-8, IL-12, IL-17, IL-21, IL-22 TNF-α, and IFN-γ [79,85,86]. The most frequently highlighted MSC-originated molecules involved in immune regulation are prostaglandin E2 (PGE2), inducible nitric oxide synthase (iNOS), and indoleamine 2,3-dioxygenase (IDO). 

Macrophages exposed to MSC-derived PGE2 are polarized toward the M2 profile and exhibit anti-inflammatory properties [87,88]. Monocyte differentiation to mature DCs is also directed toward suppression via PGE2 activity [89]. In addition, studies show that this molecule can affect T lymphocyte population recruitment, stimulating the formation of Tregs from CD4+ T cells and decreasing Th17 cell number [87,88].

Inducible nitric oxide synthase (iNOS) is an enzyme responsible for the production of nitric oxide (NO) [79,87]. Studies showed that stimulation of MSCs with IL-1α, IL-1β, and IFN-γ leads to upregulation of NO secretion [88]. NO contribution to immune system suppression includes the reduction of T-cell proliferation and Th1 and Th2 cytokine production, with the induction of apoptosis of activated T cells [79,85].

IDO-mediated enzymatic immune regulation is based on tryptophan depletion as the enzyme catalyzes the dissociation of tryptophan to N-formylkynurenine [79,87]. Tryptophan plays a crucial role in T-cell proliferation, causing metabolic changes and activation of stress signals, which are involved in Th17 differentiation inhibition and stimulation of Treg proliferation [79,89]. Furthermore, metabolites of tryptophan are more cytotoxic to CD4+ Th1 and CD8+ T cells than to Th2, resulting in a polarization of the lymphocyte population toward the Th2 profile [79]. Moreover, IDO is involved in NK cell activity suppression and macrophage polarization to immunosuppressive form [88]. In comparison to iNOS, the IDO immunosuppressive properties are more noticeable in human cells than in rodents [87].

Other often mentioned molecules of MSC origin are TGF-β, TSG-6, IL-10, CCL2, HGF, PD-L1/2, HLA-G, and HO-1, along with enzymes CD39 and CD73, which are responsible for ectonucleotide cleavage and will be discussed in detail below [79,85,87].

### 5.2. Purinergic Signaling of MSCs

**Figure 1:**
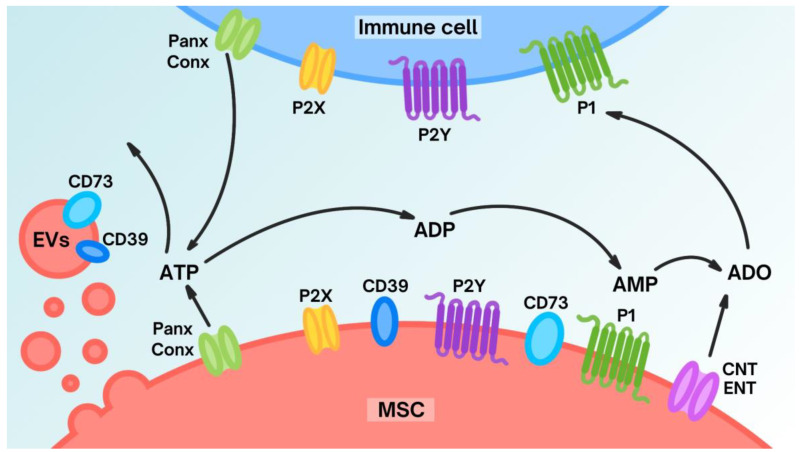
An overview of purinergic enzymes and receptors present on the surface of MSCs and immune cells and their contribution to the regulation of ATP/Ado balance in the extracellular environment [data compilation from [99,113].

MSCs have been recognized to sense the microenvironment and actively regulate the processes occurring inside it, predominantly through the secretion of various bioactive molecules, including but not limited to purine nucleotides and nucleosides [78]. The data in the literature published in 2007–2011 [90,91,92] were among the earliest reports to unveil the nucleotide signaling contribution to stem cell physiology. An increasing number of both in vitro and in vivo studies showed that MSCs release ATP to the extracellular environment, constitutively or in response to various stimuli, causing changes in the physiology of the niche. It has also been established that cells express a wide range of purine-specific receptors and enzymes that are involved in this molecular machinery [93,94,95,96,97] (Figure 1). Through P-type receptor activation, extracellular nucleotides trigger trophic effects and regulate many processes of MSCs, including proliferation, cell death, migration, and differentiation [78,93,98,99].

The ATP–Ado balance is strictly controlled as it regulates the mesenchymal stem cell pathophysiology, controlling self-renewal or initiating the differentiation of stem cells. The cell response depends on the purine concentration, type of receptor involved, duration of the receptor activation, and conditions within the extracellular environment, the so-called “microenvironmental context”. According to several sets of data, Ado has been described to stimulate the proliferation of MSCs in contrast to the anti-proliferative activity of ATP [90,91]. In contrast to these results, some studies proved that undifferentiated human bone marrow and umbilical cord-derived MSCs respond to the increase in proliferation to micromolar concentrations of ATP in the extracellular environment [100,101]. Additionally, extracellular ATP regulates MSC migration under in vitro conditions and their homing capability in vivo [92,102]. Growing evidence confirms that MSCs are characterized by a sensitivity to ATP signaling that is different from that of specialized cells. In general, ATP can contribute to both detrimental actions at higher concentrations and/or in the acute phase and also to protective and reparative effects at lower concentrations and/or in the later recovery phase [103,104]. However, in the case of multipotent MSCs, the ATP signal is interpreted as the microenvironmental requirement for regenerative processes rather than for cytotoxic effects. The nucleotide activates the biological potential of MSCs, increasing their proliferation rate and/or differentiation efficiency, depending on the local conditions in the stem cell niche.

Purinergic signaling significantly influences the process of MSC differentiation. There is no agreement on how ATP regulates osteogenic differentiation as this nucleotide was described to increase the ALP activity, osteocalcin protein expression, and matrix mineralization [105], as well as limit osteogenesis by decreasing ALP activity and mineralization [106]. Regardless of the induced effects, extracellular purine nucleotides are accepted to influence osteogenesis in human MSCs through the P2X5, P2X6, P2X7, P2Y1, P2Y2, P2Y4, and P2Y14 receptors, which are described as pivotal in this process [107,108]. For example, P2Y2 and P2Y4 receptor activation with ATP leads to suppression of mineralization processes and reduces MSCs’ ability to differentiate toward bone cells, whereas P2X7 receptors are commonly noted to play a pro-osteogenic function in MSC differentiation [109]. Similarly, in the case of chondrogenic differentiation of MSCs, some sets of results reveal that micromolar ATP concentrations enhanced cartilage regeneration, while others claim that chondrogenic processes are hampered in the presence of ATP (for an extensive review, see [108]). In a more recent report, Corciulo and collaborators [110] state that ATP is a molecule important for cartilage homeostasis, but they also highlight the role of its metabolite, Ado, in regulating the chondrocyte physiology and pathology. Additionally, adenosine receptors A1R, A2AR, and A2BR are associated with osteo- and adipogenic differentiation pathways. In the early stages of bone cell differentiation, the A2BR is upregulated, and its activation leads to the expression of genes involved in osteogenesis [111]. The same receptor is also associated with the downregulation of chondrogenesis of MSCs [112]. On the other hand, A2AR regulates both processes: in osteogenesis, it is responsible for late maturation phases, but in adipogenesis, it participates in the stimulation of marker gene expression [111]. The detailed mechanisms of adenosinergic regulation of MSC fate were recently discussed in the review paper by Galgaro et al. [99].

### 5.3. Release of Extracellular Vesicles

Purinergic signaling influencing the niche of MSCs is executed mainly through indirect paracrine activity based on MSC secretome. The secretome can be divided into two fractions: (1) soluble compounds and (2) extracellular vesicles (EVs), which can be divided further into apoptotic bodies, microvesicles, and exosomes. The soluble fraction mainly consists of cytokines, as well as growth factors and some enzymes [81]. Within the vesicular fraction, microvesicles and exosomes constitute a powerful tool in paracrine communication. These two types of lipid-bilayered structures are strongly involved in the overall signaling of MSCs, through which they regulate, inter alia, the immune system. Nevertheless, more studies are required on this topic to better understand this phenomenon [114,115,116]. One of the limitations of further research is the difficulty of EV isolation (especially exosomes), which is underlain by their small nanometric sizes and heterogeneity of EVs. Only recently has some research data linked the extracellular vesicles with certain aspects of purinergic signaling by having ATP and/or Ado in their lumen, as well as active ectonucleotidases and receptors on the membrane surface [117,118]. The ectonucleotidase activity of CD73 is often present in EVs and even mentioned as an EVs’ surface marker. The concept of adenosine-generating vesicles released by MSCs and contributing to immunomodulation seems to be a promising therapeutic solution [118]. However, there is still a lack of information on whether and how nucleotides affect the secretion of extracellular vesicles by MSCs. Studies conducted on other cell types, such as macrophages, microglia, dendritic cells, and cancer cells, indicate that ATP acting through activation of the P2X7 receptor is an efficient inducer of vesicle release [119,120,121]. Enhanced production of extracellular vesicles would be beneficial for novel cell-free therapies where secreted products are used rather than cells [122].

MSC-EVs are a perfect candidate for ASD therapy as their small size ensures their ability to cross the blood–brain barrier. Although the exact mechanism has not been identified, some research indicates a possible role of endocytosis via brain microvascular endothelial cells (BMECs) [123]. Moreover, studies indicate that EVs show homing capabilities to the inflamed regions of the pathological brain where they are uptaken by neurons [123]. Homing to the specific sites is likely caused by the adhesion molecules expressed on the EVs’ surface, e.g., integrins, CD29, CD44, and CD73 [123,124]. Exosomes’ ability to cross the blood–brain barrier was proved both when administrated intranasally and intravenously [125]. Accordingly, MSC-derived soluble factors and extracellular vesicles emerge as promising candidates for therapeutic use in different disturbances underpinned, e.g., with inappropriate purinergic signaling.

## 6. Role of Purinergic Signaling in ASD and Comorbidities

### 6.1. Purinergic Signaling Disturbances in ASD Etiology

**Figure 2:**
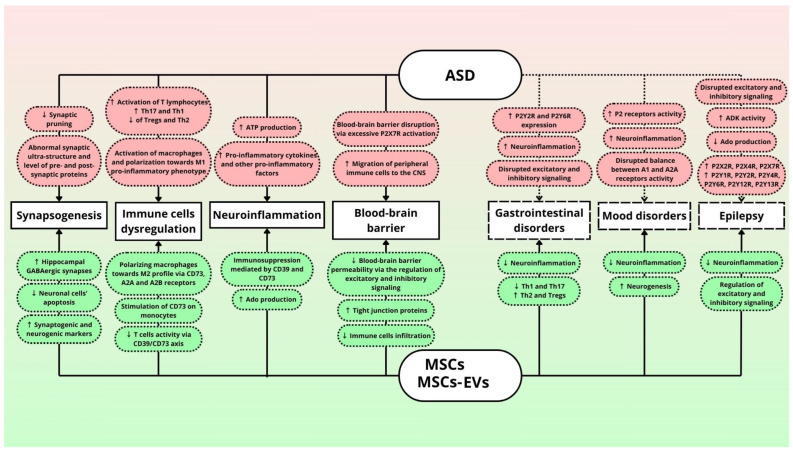
Mechanisms and processes involved in developing the symptoms of ASD and co-morbidities and the potential effects induced by MSCs and MSC-derived EVs. Up-regulated processes are marked with arrow up, down-regulated processes are marked with arrow down.

The disturbances in the processes of the migration of neurons and glia, synaptogenesis, cell proliferation, and differentiation in the early stages of brain development are confirmed to be generated by abnormal signaling through purine nucleotides and nucleosides and may lead to ASD [76,126,127,128]. One of the triggers of purinergic alterations is prenatal exposure to VPA (valproic acid), a clinical drug used for epilepsy and mood disorders, which the intake of during pregnancy largely increases the risk of ASD in children [128,129,130]. Purinergic system components and other key pathophysiological mechanisms and pathways underlying the development of ASD symptoms, as well as possible improvements by MSCs and MSC-derived MVs, are summarized in Figure 2. 

Genetic and transcriptomic analyses also confirmed the link between the abnormalities in purinergic pathways, mainly changes in receptor expression, and decreased ability in social interactions [128]. In numerous described studies, anti-purinergic therapy has been successfully applied, e.g., using suramin, a non-selective antagonist of purinergic receptors, to ameliorate the above-mentioned symptoms of ASD [128,131].

The majority of studies on animal models of ASD confirm significant alterations in P1 and P2 receptor expression at the gene and/or protein level [76,132]. An interesting case was studied by Babiec and collaborators, who focused on the changes in the expression of certain purinergic receptors in rats exposed to VPA during embryonic development [128]. Significantly elevated protein expression of A1, A2B, and A3 receptors was found, although no difference in the mRNA level of those receptors was observed [128]. Other studies highlight the importance of adenosine receptors’ cooperation in the effort to develop an effective therapeutic factor. Lewis et al. managed to achieve a significant reduction of repetitive behaviors in C58 mice only when acting simultaneously on A1R and A2AR with their agonists, CPA and CGS 21680, respectively [133,134]. Bearing in mind the capacity of MSCs and their extracellular vesicles to produce large amounts of adenosine [99], the beneficial role of these cells and/or their secretome in the course of ASD can be expected.

Broader studies were performed on the importance of P2 receptors in ASD. Significant differences in the levels of expression were described through RNA-seq, metabolomic, and transcriptomic assays. Protein levels of P2X receptors are largely modulated in ASD without any changes in mRNA expression, according to Babiec et al., who described essentially increased protein levels of P2X1, P2X2, and P2X3 receptors [76,128]. The upregulation of P2X3R itself is associated with the development of autistic symptoms, e.g., scratching behavior [76]. It is also implicated in inflammation and expressed by mast cells [135]. Furthermore, P2X4R has also been implicated in the development of many neurodevelopmental disorders [23]. Studies on P2X4R knock-out mice show phenotypic abnormalities similar to those observed in other ASD mouse models [136].

Chronic neuroinflammation observed in the pathophysiology of ASD may, through high levels of ATP in the extracellular environment, trigger an overstimulation of the P2X7 receptor, leading to its downregulation and reduced activity [76,126,128]. On the other hand, neuroinflammation may be caused by P2X7R activation, which triggers NLRP3 activity, and it further induces IL-1β, IL-6, IL-2, and MCP-1 action in the fetal brain [137]. This theory was supported by the use of NLRP3 antagonist MCC950 and neutralizing IL-1β antibody, which inhibits the development of autistic symptoms in the fetal stage [137]. Also, P2X7R antagonists, e.g., JNJ47965567, reduce behavioral ASD symptoms [76,138]. A reduced expression of P2X7R was found in the hippocampus of VPA-treated mice and in the blood of children with ASD [76,126,127]. The reduced expression of P2X7 receptors was reported in an ASD mouse model with prenatal exposure to poly(I:C), a viral mimetic used commonly to induce a controlled immune response and described to induce autism-like symptoms when administrated during pregnancy [128,139,140]. Interestingly, Horváth et al. observed that poly(I:C) manifests its role only in the presence of P2X7 receptor; an autism-like phenotype was not observed in P2X7R-deficient mice [138].

P2Y receptor levels are also anomalous in ASD. The increased expression of P2Y2, P2Y6, and P2Y8 receptors, as well as reduced expression of P2Y10 receptors, was found in the plasma of ASD children [126,127], even though not much is known about the specific role of P2Y6, P2Y8, and P2Y10 receptors in ASD. Scarce data indicate only their general association with neuroinflammation [141]. Contrarily, reduced expression of P2Y2R by 50–60% was described in the poly(I:C)-induced ASD mouse model [140]. The expression level of this receptor was improved by anti-purinergic therapy [127,140]. Reduced levels of both mRNA and proteins of the P2Y1 receptor were also determined [76,128]. Low P2Y1R expression and activity underpin abnormal neurogenesis, neuronal precursors’ differentiation, and migration, as well as disturbances in glutamate and GABA signaling [128]. At the same time, MRS2179, a selective P2Y1R blocker, suppresses microglial activation that normally leads to ATP-triggered neuroinflammation, although it does not alleviate behavioral symptoms typical for post-injury inflammation [142]. Other studies show that P2Y1 receptors may decrease the susceptibility of neurons to excitotoxicity through the reduction of glutamate receptors (NMDAs), leading to neuronal damage and oxidative stress in patients with ASD [143,144].

P2Y12R activation is linked with pathological CNS conditions and mediates a stress-induced synapse loss in the prefrontal cortex, worsening the working memory function [76,145]. P2Y12R-positive microglial cells, largely expressed in the cortex of VPA-treated offspring and formed plausibly by epigenetic changes induced by VPA, were described to secrete pro-inflammatory mediators such as iNOS, IL-1β, and TNF-α [76,145].

Besides various alterations in purinergic receptors, research also indicates a major role of enzymes metabolizing nucleotides in early brain development as well as in ASD etiology [76,127]. RNA-seq analysis was harnessed to show abnormal expression of adenosine deaminase (ADA), adenylosuccinase (ADSL), and bifunctional 5-aminoimidazole-4-carboxamide ribonucleotide transformylase/IMP cyclohydrolase (ATIC) in ASD patients [126,127]. ADSL expression was found to be reduced, while the expression of ADA and ATIC was significantly increased [126]. Although other studies report a reduction of ADA and ATIC in neurodevelopmental disorders, they agree in terms of the deficiency of ADSL and its key role in ASD [126,146]. Decreased expression of ADSL strongly affects purine metabolism and may be a reason for the reduced uric acid concentration in patients with ASD [126,147]. The concentration of uric acid was assayed in metabolomic studies and was proven to be decreased in blood and urine, which is thought to resemble the levels in cerebrospinal fluid [126,128,148]. Uric acid functions as an antioxidant; therefore, its deficiency in the CNS may lead to the development of oxidative stress and largely affect the neurodevelopmental process [126]. The lack of enzymatic activities can be, at least to some extent, amended by the enzymes of neighboring cells, e.g., MSCs homed to the appropriate niche, or by MSC-derived vesicles.

### 6.2. Purinergic Signaling Disturbances in ASD-Related Comorbidities

#### 6.2.1. Epilepsy

Epilepsy is a neurological disorder characterized by abnormal and excessive neuronal activity in the brain, resulting in reoccurring seizures [149,150,151]. Hyperactivity is a consequence of an imbalance—either excessive excitation or deficient inhibition in the brain [152]. Epilepsy affects around 0.7–1% of people worldwide [151,153]. In ASD patients, the prevalence of epilepsy ranges from 2.4% to 46% and is significantly higher for individuals with intellectual disabilities [149,153]. Epileptic seizures occur not only in childhood but also persist into adolescence and adulthood, making this comorbidity even more challenging [149].

Numerous studies indicate the role of purinergic signaling in epilepsy. Both adenosine and ATP participate in a process called epileptogenesis—the transformation of a healthy brain to a brain able to spontaneously develop seizures. Important points of epileptogenesis are, among others, inflammation, abnormal neurogenesis, and neurodegeneration [154,155]. Adenosine, when released during seizures, exerts its protective effects through A1 receptors, significantly shortening the duration and decreasing the intensity of seizures through the regulation of neuronal hyperactivity [152,154,155]. The role of A2 receptors is not fully known, although research suggests their opposite, pro-convulsive role [154]. Extracellular levels of adenosine are largely affected by purinergic metabolism, which ADK (adenosine kinase) takes part in. Significant upregulation of ADK is observed during seizures, resulting in decreased levels of adenosine and enhancing the seizure susceptibility [156].

ATP also plays a crucial excitatory role during the development and duration of seizures [155]. Increased levels of P2X2, P2X4, and P2X7 receptors have been detected in the epileptic brain [154,155,157]. Moreover, levels of P2Y1, P2Y2, P2Y4, P2Y6, P2Y12, and P2Y13 receptors are also increased [154,155]. High expression of P2X7R is also linked to the release of IL-1β, a cytokine with pro-convulsive properties, which contributes to neuroinflammation [155,157]. Research conducted on epileptic mice showed that P2X7 receptor antagonist JNJ-47965567 significantly reduces spontaneous seizures, proving the pro-convulsive activity of P2X7R [157].

MSCs have the potential ability to correct the imbalance between excitatory and inhibitory pathways in the brain through purinergic signaling, mainly by releasing the previously described anticonvulsant—adenosine. MSCs may affect the excitatory pathway, induced by excessive ATP, indirectly by modulating the inflammatory response and, therefore, reducing the neuroinflammation present in epilepsy. Adenosine is produced partially by ectoenzymes CD39 and CD73, both of which are present on the surface of MSCs [158]. It has been previously reported by Lanser et al. that the deficiency of CD39 in knock-out mice results in a disrupted ATP/adenosine ratio, which further correlates with the seizure phenotype [159].

Promising results have been described considering the use of MSCs in the treatment of epilepsy. Liu et al. showed a successful effect of autologous olfactory mucosa-derived mesenchymal stem cells (OM-MSCs) administrated in an epileptic mouse model. OM-MSC treatment was efficient in enhancing the cognitive, locomotive, and perceptive functions without any adverse effects. Results were caused by the recruitment of Treg cells to the brain, which resulted in a decrease in inflammation and rebuilding of the neural network. Inhibition of the inflammatory processes was proved by determining the level of cytokines. Pro-inflammatory cytokines (TNF, IL-1β, and IL-6) were found to be decreased, while anti-inflammatory cytokine, IL-10, was significantly increased [160]. Another study using bone marrow-derived MSCs (BM-MSCs) proved their positive effects when applied to epilepsy patients in a clinical trial. A significant decrease in the seizure count was observed [161].

#### 6.2.2. Mood Disorders

Mood disorders (otherwise known as affective disorders) are a group of psychiatric disorders characterized by persistent changes, elevation or decline, in one’s mood. The two most common disorders, MDD (major depressive disorder) and bipolar disorder, affect over 350 million people worldwide [162,163]. The incidence of depressive disorders is 16%, while bipolar disorder is 5% [162]. Mood disorders are one of the most prevalent comorbidities of ASDs [164,165]. The prevalence rates for depression and bipolar disorder are much higher compared to the general population and rise to 23–37% and 6–40%, respectively [165]. Coexisting psychiatric disorders contribute not only to reduced quality of life for patients but also for their caregivers [164].

Purinergic signaling regulates key processes involved in affective disorders such as mood, cognition, social interaction, etc. [166,167]. Several in vitro studies point to ATP and adenosine as molecules involved in the etiology of mood disorders [166].

It has been shown that depressive mood might be triggered by ATP released from astrocytes and by the overactivity of P2 receptors [166]. Administrating P2 receptor antagonists alongside antidepressants seems to further reduce depressive symptoms in mice [167]. eATP acting extensively on P2X7 receptors and showing pro-inflammatory activity seems to be the key factor implicated in the etiology of depression [166,168]. P2X7R knockout mice display anti-depressive effects [166,167]. Studies on mice proved the relevance of NLRP3 inflammasome in depressive behavior, therefore supporting the neuroinflammation theory of depression [168]. This theory is further encouraged by P2X7 antagonists that inhibit the P2X7-NLRP3-IL-1β pathway [168]. The extracellular vesicles of MSCs, as described above, are already known to reduce neuroinflammation—one of the key factors implicated in the etiology of mood disorders [169].

The most implicated adenosine receptors in the etiology of mood disorders are A1 and A2A [166]. First, studies researching the role of these receptors in depression utilized their non-selective antagonist—caffeine. Moderate doses of caffeine showed anti-depressant effects and reduced suicide risks, while high doses induced episodes of mania [167]. Increased activity of those receptors in rats, as well as A1R knockout mice, are linked with enhanced depressive symptoms [166,167]. Overall, it seems that the balance between A1 and A2A receptor activity is a key factor in the proper release of neurotransmitters [167].

Several studies have shown the ability of MSCs to alleviate the depression symptoms directly. Encapsulated mesenchymal stem cells act through the secretion of neurotrophic factors, affecting signaling pathways linked with depression as well as neurogenesis stimulation [170]. Interestingly, research shows the interaction between neurotrophic factors and purinergic signaling pathways. Both P2X7 and P2X4 receptors have been linked to BDNF (brain-derived neurotrophic factor) regulation. Furthermore, the P2X4 receptor has also been implicated in depression—ivermectin, an allosteric modulator of the P2X4 receptor, is proven to worsen depressive behavior [166]. ATP, through P2X7 receptors, induces IL-1β release. This further activates the P2X4 receptor, which directly stimulates the release of neurotrophic factor—BDNF [171]. Studies show that the downregulation of BDNF seems to be related to depressive behavior [172]. 

Another study was performed on CUMS (chronic unpredictable mild stress model) mice [173]. hUC-MSCs (human umbilical cord mesenchymal stem cells) were administrated for 4 weeks once a week, and a significant amelioration of depressive behavior was observed [173]. Several doses of hUC-MSCs, through complement C3 and C3a signaling modulation, altered microglia M1/M2 polarization, which resulted in increased levels of anti-inflammatory factors, therefore alleviating inflammation [173]. Complement C3 and C3a have been implicated in the pathophysiology of MDD (major depressive disorder)—increased levels of both elements were found in the plasma of medication-free patients [174]. Furthermore, a link has been found between purinergic signaling and C3. Apparently, suramin, an antagonist of P2 receptors, inhibits complement activation, providing further evidence for the link between purinergic signaling, neuroinflammation, and mood disorders [175] (Figure 2).

#### 6.2.3. Gastrointestinal Disorders

Gastrointestinal disorders (GIDs) include a wide variety of conditions with varying levels of severity [176]. Some examples of GIDs include inflammatory bowel disease, irritable bowel syndrome, acute diarrhea, or functional constipation [176]. A wide range of the causes of the disturbances are observed, including viral and bacterial infections, transmission through food or water, as well as secondary consequences of antibiotic therapy [176]. Gastrointestinal disorders are a common comorbid condition in ASDs, although the mechanisms of action are mostly unknown [177]. Prevalence rates, reported in the range from 9% to over 90%, are also challenging to elucidate [177].

One of the most researched gastrointestinal disorders present in patients with ASD is IBD (inflammatory bowel disease). As the name suggests, IBD is closely correlated with inflammation in the gastrointestinal tract. Similarly to epilepsy, a key factor linked to the pathogenesis of IBD is the imbalance in the ATP/adenosine ratio [178].

P2X7 is the most crucial receptor involved in IBD. It has already been shown to trigger proinflammatory cytokine release from the immune cells when activated by ATP [179]. Recent studies have shown that P2X7R knockout mice, when compared to WT mice, have a much lower susceptibility to colitis induced by chemical agents [180]. P2X7R activation leads to the stimulation of Panx1 (pannexin-1) channels and inflammasome, which in turn triggers neuronal death [178]. Interestingly, it has been recently observed that oxidative stress triggers opposing processes when inducing the Panx1 opening. On the one hand, an increase in ROS leads to ionic dysregulation, which results in cell death. On the other hand, Panx1, when opened, secretes ATP into the extracellular environment, where it binds to the P2Y12 receptors present in microglia. Increased microglia contacts exert neuroprotective processes [181]. Inhibition of Panx1 and P2X7R activity significantly reduces the release of proinflammatory cytokines (e.g., IL-1β), therefore also reducing inflammation [178,179]. P2X7R, when activated, has also been observed to induce the release of proinflammatory cytokines, chemokines, and leukotrienes by mast cells. Secreted molecules trigger the migration of neutrophils to the area of inflammation [178].

P2Y receptors have also been implicated in the pathogenesis of gastrointestinal disorders. The activation of the P2Y2 receptor increases the expression of ICAM-1, which triggers macrophages’ migration and adhesion [178]. P2Y6R has also been described as promoting neutrophils’ migration through the regulation of CXCL8 expression [178]. The upregulation of both P2Y2 and P2Y6 receptors has been observed in a rat model of TBNS-induced colitis [179].

The immunomodulatory abilities of MSCs have been of significant importance in the treatment of IBD. MSCs downregulate Th1 and Th17 while upregulating Th2 and Treg cells. By the secretion of IDO (indoleamine 2,3-dioxygenase), MSCs may also promote the differentiation and maturation of Th2 cells [182]. Th1/Th2 ratio imbalance is largely responsible for the inflammation responses—Th1 cells cause excessive inflammation in the organism, suppressed by Th2 cells secreting anti-inflammatory factors [57,182]. Similarly, the Th17/Treg ratio is also crucial. Th17 plays a pro-inflammatory role, while Tregs trigger anti-inflammatory responses [57,182]. As described earlier, purinergic signaling greatly contributes to the MSCs’ immunomodulatory function (Figure 2). 

Some promising results have recently been obtained when treating patients diagnosed with Crohn’s disease with a few doses of UC-MSCs administered intravenously. Only mild side effects were observed [183]. Another study conducted by Barnhoorn et al. proved the efficacy of Crohn’s disease treatment by allogenic BM-MSCs even after 4 years. At the same time, no serious adverse effects of the treatment were observed [184].

## 7. Role of Mesenchymal Stem Cells in ASD Treatment

### 7.1. Purinergic Implications of MSCs in Neuroinflammation

Among many MSC mechanisms of action, purinergic signaling plays omnipresent functions in immunoregulation, neurogenesis, and brain physiology modulation [81]. These processes are also depicted in Figure 2. In this section we present possible pathways and mechanisms through which MSC-derived purinergic machinery can participate in amelioration of ASD-related neuroinflammatory events. 

In the extracellular environment, ATP functions as one of the damage-associated molecular patterns (DAMPs), in which elevated concentration leads to the activation of immune system cells and the production of pro-inflammatory cytokines and factors [185]. Also, ATP stimulation of MSCs results in the secretion of various enzymes and factors, including COX-2, IDO, and iNOS, which stimulate the pro-inflammatory activity of the immune system [186,187]. The treatment of murine MSCs with TNF-α and IFN-γ indicated that both pro-inflammatory factors upregulate the expression of many molecules involved in immune regulation, such as COX-2, PGE-2, hepatocyte growth factor (HGF), and IDO, as well as programmed death-ligand 1 (PD-L1) on the MSCs’ surface [188]. Another study has reported that bone marrow MSCs stimulated with IFN-γ and ATP have higher expression of IDO compared to non-treated MSCs or those stimulated only with IFN-γ. It has been tested by flow cytometry and indirectly by measuring kynurenine, a product of tryptophan metabolism [189]. The application of apyrase, an enzyme degrading ATP or P2X7 receptor antagonist, led to the abolishment of IDO expression and kynurenine production [189]. A neuroinflammatory state, commonly correlated with the presence of extracellular ATP, may also activate MSC-dependent immunoregulation and ameliorate the harmful immune activation during ASD. Furthermore, conditioned media from ATP- and IFN-γ-stimulated MSCs have the ability to inhibit the proliferation of peripheral blood lymphocytes [189]. Alternatively, conditioned media with ATP and other pro-inflammatory factors could potentially be used as a cell-free therapy.

Numerous studies highlight the possible role of the extracellular nucleotide balance and its regulation by MSCs’ ecto-enzymes in the therapy of inflammatory diseases [190,191]. CD39 and CD73 are considered the main enzymes involved in MSC-mediated immunosuppression [78]. These two proteins are able to switch the pro-inflammatory environment toward an anti-inflammatory one via catalyzing the transformation of ATP to AMP and AMP to Ado, respectively [78]. The enzymatic hydrolysis of ATP and AMP by MSCs from different sources was confirmed by many research groups [93,96,192]. Obtained amounts of Ado were related to added ATP, and the inhibition of CD39 resulted in the inhibition of the whole pathway, which strongly reduced Ado concentration [192]. The presence of the ecto-enzymes is not only limited to the cellular surface, but they appear on extracellular vesicles as well [99,118]. CD73 activity has been shown on the MSC-derived extracellular vesicles in many studies. On the other hand, CD39 is still to be elucidated as there is a high possibility to find it on the surface of vesicles [99,118]. Migration of extracellular vesicles in diseases with ongoing inflammation has been presented for bone marrow MSCs exosomes that were intranasally applied to mice. According to the findings, MSC exosomes were migrating to the cerebellum and prefrontal cortex of BTBR murine models, regions strongly associated with ASD, and stayed there up to 96 h after application. This result has not been observed in the control, a healthy group in which exosomes stayed in the administration area and vanished after 24 h [193]. Additionally, authors found a correlation between the migration of exosomes and inflammation in the cerebellum, which was indicated via staining of CD11b, a marker of activated microglia [193].

As discussed below, the neuroinflammatory effects triggered by immune cells, astrocytes, and microglia require the contribution of purinergic receptors and signaling compounds, which further result in the secretion of pro-inflammatory molecules (see Figure 2). MSCs have the ability to counteract this activity by regulating the excitatory/inhibitory transmission and, therefore, reducing neuroinflammation present in ASD. Thus, MSCs’ anti-inflammatory functions fit perfectly as the potential treatment option.

### 7.2. MSCs Influence on Immune Cells

The pathology of ASD is correlated with abnormalities among immune cells, suggesting the opportunities for possible application of MSC-derived purinergic regulation in amelioration of the symptoms (summarized in Figure 2). Monocytes and macrophages are cells with a strong association with innate immune response and inflammatory processes [194,195]. Those cells function through the monocyte/macrophage axis, in which monocytes are stimulated by different immune regulating factors that differentiate into specific macrophage profiles that are able to modulate their cellular niche [194,195]. Some research indicated the ability of CD73 on MSC exosomes to polarize macrophages toward the M2 anti-inflammatory profile [196]. This effect was manifested via upregulated expression of macrophages’ M2-associated genes, including CD206, CCL24, CCR1, Arg1, IL-1RN, IL-10, IL-13, and PPARγ, with no effect on the expression of iNOS, TNF-α, IFN-γ, CD80, CCL5, IL-1β, IL-12β, and IL-6 on non-activated macrophages. The contribution of CD73 in this pathway was confirmed by using the CD73 inhibitor, which abolished the expression of those genes [196]. Furthermore, it was proven that this regulation is strongly dependent on the activity of adenosine A2A and A2B receptors and the AKT/ERK phosphorylation pathway [196]. Another study provides evidence about MSC stimulation of CD73 expression on monocyte cells. Monocytes co-cultured with MSCs exhibited upregulated mRNA expression of M2 profile markers, including CCL18, TGM2, CD206, and CD163, together with elevated expression of CD206 and CD163 on the cell surface and secretion of IL-10 [197]. After direct co-culture of MSCs with monocytes, there was an increased expression of CD73, while no effect on CD39 was observed, but not with conditioned medium and during Transwell™ assay, which indicates the requirement of direct contact between cells. Additional functional tests of CD73 activity of monocytes were also performed, confirming this ectonucleotidase activity [197]. Furthermore, overexpression of CD73 on monocytes was proven in in vivo trials of swine models of infarcted myocardium, a condition correlated with elevated immune activation around the injury, in which MSCs were transplanted as the bioactive compound [197]. While ASD gains more and more evidence of monocyte and macrophage activation among affected individuals, the two mentioned articles point out a possible direction of future studies, in which MSCs, through purinergic signaling, can directly or indirectly switch immune cells profile toward anti-inflammatory.

Besides monocytes and macrophages, the T lymphocyte pathologies among ASD patients are also reported. The extracellular nucleotide profile regulation by MSCs and suppression of activated T lymphocytes confirmed that MSCs have the ability to inhibit T-cell activity via purinergic signaling [198,199,200]. The activated T lymphocyte proliferation was reduced by 50% to 75%. The CD39/CD73 axis, which leads to the production of adenosine and stimulation of A2A receptors, is indicated as one of the main mechanisms underlying T-cell suppression, proven by the usage of proper inhibitors of ectonucleotidases and adenosine receptor antagonists [198,199,200]. According to Saldanha-Araujo et al., the MSCs co-cultured with T cells in an inflammatory environment produce up to two times more adenosine than those cultured alone [199] and stimulate the CD39 and CD73 expression on T lymphocytes [199,200]. It is worth mentioning that in those studies, tests were performed both on murine cells [198] and on cells sourced from humans [199,200], indicating the stability of this process across species. All of those articles bring evidence of the ability of MSCs to regulate inflammatory disturbances among T cells in ASD. The application of those cells can ameliorate abnormality in ASD patients’ Treg lymphocytes by reducing pro-inflammatory T-cell activity.

Microglia and astrocytes, together or alone, contribute to many physiological processes in the brain, which include the neurodevelopment during the early stages of life, synaptic pruning, and the formation of new neural transmission pathways or proper functioning of blood–brain barrier (BBB) [74,75]. Synaptogenesis in humans starts in the fetus and continues until reaching stability in infancy or adulthood [201]. A disruption of synapses in ASD is implicated as early as in the prenatal period, making it a crucial process in the fetus’ development [201]. Studies on human post-mortem brains prove the dysregulated process of synaptic pruning—maturation of necessary synapses and elimination of excessive ones, which results in brain overgrowth [201]. Insufficient pruning in ASD might be partially explained by microglial abnormalities, as microglia are physiologically responsible for the promotion of neurogenesis and synaptic pruning in the organism [202]. Microglia, by the secretion of pro-inflammatory molecules, may promote neuroinflammation and, consequently, synaptic changes in ASD patients’ brains [63]. The role of maternal immune activation in the development of synaptic dysregulation, including abnormal synaptic ultra-structure and changes in the level of pre- and post-synaptic proteins, was reported [203]. Moreover, VPA induces pathological alterations in synapse ultrastructure, both directly on VPA-exposed neurons as well as indirectly through the VPA-exposed astrocytes [63,204,205]. The astrocytes exposed to VPA disrupt the excitatory–inhibitory balance in the CNS through the impairment of inhibitory synaptic formation and transmission. The co-culture of rat MSCs and hippocampal neurons proved MSCs’ beneficial effect on synaptogenesis. Mesenchymal stem cells significantly increased the hippocampal GABAergic pre-synapses in vitro [206]. Moreover, a decreased apoptosis of neuronal cells in the hippocampus in the presence of either hUMSCs or hAD-SCs was observed together with an increased expression of synaptogenic and neurogenic markers—synaptophysin and GAP43, respectively [207].

The neuroprotective effects of extracellular vesicles derived from MSCs were observed in a hypoxia–ischemia mice model. MSCs-EVs successfully inhibited neuroinflammation by reducing osteopontin expression in microglia and infiltrating monocytes/macrophages. Moreover, MSCs-EVs attenuated synaptic damage and increased synaptic protein expression [208]. Interestingly, osteopontin expression is speculated to be linked with long-term brain damage [208]. Excessive levels of osteopontin during neuroinflammation are underpinned directly by the increased level of ATP. P2Y1 antagonist, MRS2179, significantly inhibits this process [209]. Therefore, MSCs could affect disrupted synaptogenesis in ASD through various pathways directly by restoring the proper physiological synaptic protein levels or indirectly by affecting neuroinflammation—reducing the concentration of extracellular ATP and pro-inflammatory cytokines or reducing the level of osteopontin.

### 7.3. Purinergic Implications of MSCs in Blood–Brain Barrier Permeability

As mentioned earlier, MIA during pregnancy disrupts the blood–brain barrier formation, potentially leading to neurodevelopmental disorders in the fetus. In physiological situations, BBB is highly selective, which is advantageous when it comes to limiting the spread of inflammation to the brain [210]. The BBB mainly consists of endothelial cells as well as neurons, astrocytes, microglia, and oligodendrocytes—cells that possess purinergic receptors and secrete purines [210]. Furthermore, several studies describe the modulation of BBB permeability through purinergic pathways. Excessive levels of ATP acting through the P2X7 receptor are known to increase BBB permeability and the migration of peripheral immune cells to the CNS [211]. Thus, the administration of P2X7 receptor antagonists leads to an increased claudin-5 expression—a protein that builds the tight junctions between the endothelial cells and, therefore, lowers the permeability of the BBB [212]. Potential MSCs’ therapeutic effects can also be supported by the catabolism of ATP via the CD39 and CD73 ectonucleotidases [213]. The formation of adenosine seems to be beneficial as the role of Ado in the BBB permeability regulation was proven [214]. However, the importance of maintaining a proper balance between excitatory and inhibitory signaling must be once again highlighted, as an increase in blood–brain barrier permeability under the influence of A2A receptor agonists also occurs [215]. The inhibition of CD73 and the blocking of the A2A receptor limit the infiltration of lymphocytes into the CNS [216]. Therefore, MSCs, through their fine-sensing and regulatory abilities, might lead to the tailored excitatory and inhibitory signaling ratio and further maintain the low permeability of the BBB. MSCs may also regulate the blood–brain barrier permeability directly. Park et al. proved that the stabilization of the BBB and inhibition of neutrophil infiltration through MSC treatment led to the upregulation of tight junction proteins, enhanced filament density in endothelial cells, and modulation of astrocytic endfeet. Moreover, MSCs decreased inflammatory processes by downregulating IL-1β production in microglia and upregulating IL-10 levels in astrocytes [217] (Figure 2).

### 7.4. Current MSC Applications in ASD Treatment in Animal Models and Clinical Trials

The concept of “medicinal signaling cells” reflects MSCs’ ability to modulate many physiological functions of the neighboring niche via their secretome. Thus, the MSCs became a promising tool in the regulation of cellular aberrations present in many diseases, including those with dysregulation of the immune system. The application of MSCs in the treatment of ASD is quite a new idea, which is gaining more and more interest among researchers and has been investigated in recent in vivo studies.

Studies on ASD animal models, e.g., BTBR mice displaying autism-relevant behaviors, bring hopeful outcomes of MSC usability. Amelioration of symptoms of BTBR mice, such as enhancing mice socialization, memorizing skills, and tendency to explore, was confirmed after intravenous transplantation of human bone-marrow MSCs into tail blood vessels [218]. The gut commensals profile was also tested, and results indicate improvement within its composition, reminding those of the control group [218]. Similar results were reported by Segal-Gavish and his group using human MSCs transplanted into lateral ventricles of BTBR mice [219]. The three main symptoms, i.e., the occurrence of repetitive behaviors, socialization, and ability to adapt, have been tested at least in two different tests, proving the beneficial effect of MSC transplantation [219]. Furthermore, a positive effect on neurogenesis was observed: the concentration of Ki67+ cells and doublecortin+ cells was elevated in the hippocampus [219].

ASD mice from mothers treated with valproic acid during gestation were also stimulated with bone marrow MSCs via injection to the lateral ventricle [220]. Apart from the amelioration of social and behavioral impairment in these animals, the neurogenic processes were also monitored in this set of experiments. Although no differences in the number of doublecortin+ cells were recorded between groups, the ratio of innate to mature doublecortin+ cells was significantly decreased in MSC-treated groups and those without exposure to valproic acid [220].

Not only were cells used for modulating ASD symptoms, but their secretome was also rich in extracellular vesicles. Intranasal dosing of MSC-derived exosomes was proved to ameliorate BTBR mice’s ASD phenotype [221]. Similar to the above-mentioned research, mice spent more time in social interactions and less time in repetitive behaviors; they also presented closer to normal vocalization, and female mice cared more about their offspring than before treatment [221]. In addition, the same research group also tested another ASD mouse model, Shank3B, obtaining results similar to the previous study. Additionally, it was observed that MSC exosomes stimulate the expression of GABA Ra1 in the prefrontal cortex, which corresponds to the amelioration of GABAergic dysfunction in ASD patients [222].

The presence of different possible treatments using MSC cells, conditioned media, or isolated vesicles raises uncertainty about which of these methods is better for future application. Efficiency and safety issues are also strongly combined with ways of administration of MSCs or their EVs [223]. There are several possible options for the application of MSCs, which, depending on the delivery area, can be categorized as those with systemic or local ways of action. Among the methods of MSC administration used in ASD treatment are injections such as intravenous, lateral ventricular, intrathecal, or intranasal application. As has been presented above, all of these methods were applied in ASD with promising efficiency and with no recorded serious side effects. Nevertheless, the scientific literature shows concerns and minuses regarding the use of some techniques. Intravenous injection of MSCs is one of the most often used methods of cell administration [122,224,225]; however, some studies indicate that MSCs do not reach the final desired organ with a primal concentration. Using this method leads to the accumulation of MSCs and their debris in the lungs and then their redistribution to the liver, kidneys, and spleen [122,225]. Problems with reaching the final destination could be resolved by using direct application to lateral ventricles. However, this method is characterized by higher-than-usual invasiveness due to the requirement of puncturing the skull. In addition, there is still limited information about the fate of cells after transplantation using this procedure [226]. Intrathecal administration is based on the injection of MSCs in the upper region of the spine [122,224]. This technique is a valuable substitute for lateral ventricle injection because it directs MSCs toward central nervous system distribution with limited systemic administration. Furthermore, there is evidence that MSC translocation from the spine to the brain depends on the dose of transplanted cells, which should be closely investigated during the development of future therapeutics [224]. Intranasal administration of MSCs is the newest established method. Due to its safety, easy protocol, and efficiency, it is becoming more and more popular. After the application of cells in aerosol into the nose, they reach the olfactory bulb and then cerebrospinal fluid, eventually ending in a particular region of the brain [122,226]. This technique is commonly used in cell and drug administration for central nervous system-associated diseases. In addition to cell-based therapies, cell-free approaches have recently attracted more and more attention due to their crucial advantages. Administrated EVs pass the blood–brain barrier more easily than MSCs, which successfully enhances their therapeutic efficacy. This technique is generally considered safe—it does not pose the risk of immunogenicity and tumorgenicity. Moreover, it is easier to store, transport, and produce EVs in large quantities when compared to MSCs. Currently, EVs are mostly delivered systemically, although this route causes the dispersion of EVs and their short residence time. More efficient ways are still being investigated [123,227]. Intranasal administration of bone marrow MSCs and their conditioned medium in the treatment of rats from valproic acid-exposed mothers gave evidence of behavior improvement, but only in some of the tests, whereas in the others, including repetitive behavior and passive avoidance, there were no significant differences between those two types of treatment [228]. Cellular therapy gives better results in immune regulation than using a conditioned medium and significantly decreases the concentration of pro-inflammatory cytokines IL-6 and IL-1β and stimulates IL-10, which is responsible for anti-inflammatory response [228]. Additionally, the presence of microgliosis in brain tissue was also investigated. Since valproic acid stimulates the expression of Iba-1 in the hippocampus, the bone marrow MSC-treated group exhibited a significantly lower concentration of this molecule [228].

In another set of experiments, mononuclear cells from human cord blood and MSCs from the umbilical cord were transplanted intravenously (one infusion) and intrathecally (three infusions) to ASD patients. This study enrolled 37 patients between 3 and 12 years old, all with confirmed ASD [229]. Only a few patients had a slight fever, which eventually did not require any medical actions. There were also no serious side effects reported during this treatment [229]. Patient behavior was tested using three methods: Childhood Autism Rating Scale (CARS), Clinical Global Impression (CGI), and Aberrant Behavior Checklist (ABC). All of them indicated improvement in ASD symptoms [229]. The safety of the treatment was also tested in a study by Sun et al. In this research, 12 children with ASD in the ages 4–9 were treated with one to three doses of umbilical cord MSCs transplanted intravenously [230]. Their health parameters were measured at day one and after 6 months after injection. Additionally, data about their condition were repetitively checked via questionnaires during treatment and 6-12 months after the last dose [230]. Two of the twelve participants had adverse responses to infusion, which required medical intervention. The authors postulated that this could be due to using DMSO for cryopreservation of cells. Otherwise, transplantation was safe for participants. Finally, half of the patients had amelioration of ASD symptoms in at least two out of three applied methods [230].

## 8. Concluding Remarks and Future Perspectives

Mesenchymal stem cells offer much more than enhanced regeneration to the treatment modalities. Their ability to home to the site of injury, as well as their ability to release many active soluble compounds, extracellular vesicles, and ecto-enzymatic activities on their surface, make them promising therapeutic tools in many disturbances. Dysregulation of the immune system with a bias toward neuroinflammation, underlying the development of ASD, requires efficient immunosuppressive and anti-inflammatory therapeutic strategies. MSCs, as actively signaling cells with strong immunomodulatory properties, fit this approach perfectly. Among many of MSCs’ mechanisms of action, purinergic signaling plays omnipresent functions in both immunoregulation and brain physiology modulation, thus creating a promising perspective. There are also doubts and limitations of such a therapeutic approach. One of them is the heterogeneity of MSCs; thus, careful testing of what doses of MSCs and at what frequency they have to be administered is necessary. We lack the knowledge of whether long-term structural and functional changes in the brain tissue will be possible to stop or reverse. Efficiency, safety, and, most importantly, the final outcomes can differ between tested model organisms and human patients. Nevertheless, MSCs have the ability to reduce neuroinflammation through the purinergic machinery. We anticipate that a better understanding of purinergic signaling may help to restore neurotransmitter balance, to reduce neuroinflammation, and to improve overall brain function in individuals with ASD.

## Data Availability

Data are contained within the article.
